# Why do people spend money to help vulnerable people?

**DOI:** 10.1371/journal.pone.0213582

**Published:** 2019-03-15

**Authors:** Luminița Pătraș, Vicente Martínez-Tur, Esther Gracia, Carolina Moliner

**Affiliations:** University of Valencia, Research Institute in Personnel Psychology, Organizational Development and Quality of Working Life Research Unit in Work and Organizational Psychology (IDOCAL), Valencia, Spain; Middlesex University, UNITED KINGDOM

## Abstract

Prosocial spending has been linked to positive benefits for individuals and societies. However, little is known about the precursors of prosocial spending directed to vulnerable people. We experimentally tested the effect of a first exposure to a prosocial donation decision on subsequent prosocial spending. We also examined the direct links from eudaimonic well-being beliefs (contribution-to-others and self-development) to prosocial spending, as well as the interaction between these beliefs and autonomy in predicting the money given. A total of 200 individuals participated in the study. Results showed that, compared to two control groups (“totally self-focused” and “no first-exposure”), an initial exposure to a prosocial donation decision increases subsequent prosocial spending. In addition, we observed an anchoring bias from the initial prosocial donation to subsequent prosocial spending. Regression analyses also confirmed the existence of a positive significant relationship between contribution-to-others beliefs and prosocial spending. Finally, we observed a significant interaction between autonomy and self-development well-being beliefs, such that autonomy strengthens the link from self-development beliefs to prosocial spending. In general, our results confirmed the significant role of exposure, anchoring, autonomy, and well-being beliefs in predicting the money spent to help vulnerable people.

## Introduction

Self-interest, or the maximization of utility, has been a consolidated economic assumption for at least a century [1]. It is also present in psychological approaches such as the pleasure principle, proposed by Freud [2], or social learning theories that emphasize positive reinforcement from the environment [3]. However, human behavior cannot be restricted to individual self-interest. Humans have been able to create complex interactions and societies where the maximization of one’s interests is combined with moral bonds [4] and motivations such as solidarity and cooperation [5]. In fact, prosocial behaviors are present in human evolutionary history [6]. The research by Kelly [7] examining hominids during the 2.9-million-year Paleolithic time span demonstrated that help and cooperation allowed human groups to flourish in different regions of the globe. Prosocial behaviors are also observed in children and chimpanzees [8], indicating that these behaviors are part of human nature and, probably, also a characteristic of other living beings.

Prosocial behavior is a general concept that refers to different types of actions to benefit others [9]. Prosocial behaviors include both small helping actions and more formalized prosocial behaviors, such as volunteering, donating blood, or giving to charity [10]. Prosocial behavior has often been used interchangeably with altruism. There is a research tradition that focuses on the measurement of prosocial behavior and altruism and their antecedents. The use of the Dictator Game as a measure of altruism is especially noteworthy [10,11]. However, they are distinct concepts [12]. Whereas altruism is considered the motivation to help others out of absolute regard for others’ needs, prosocial behavior refers to a pattern of activity to help others, regardless of the helper’s motivation [13].

The concept of prosocial spending was introduced as a specific form of prosocial behavior, and it is defined as spending money on others as opposed to on oneself, usually in the form of gift giving or charitable donations [14]. Spending money on others has important benefits for communities and society at large by building strong local communities, promoting bonds of trust among neighbors [15], and sustaining entities that provide critical education, health, arts, environmental protection, and disaster relief services. In 2015, individual donations as a percentage of gross domestic product hovered around 2.1% in the U.S. [16]). The proportion of people giving to charity has also grown across European countries in 2014 and 2015 (38%) [17]. Donations focus on causes such as helping children and young people, healthcare, poverty reduction, international cooperation, and community and social aid. Prosocial spending is also beneficial at the individual level, not only for the receiver, but also for the giver. From the giver’s perspective, prosocial spending contributes to his/her happiness and well-being [14,18–20] and increases positive emotions [20]. These benefits for the giver have been observed in different countries and cultures, to the extent that the “warm glow of prosocial spending” has been proposed as a psychological universal [19].

Due to the positive benefits of prosocial spending for both individuals (giver and receiver) and society in general, it is relevant to diagnose the factors predicting prosocial spending. Previous research efforts have concentrated on constructs such as compassion and religious beliefs, with somewhat contradictory results [21,22]. The current research study focuses on aspects that are closer to the specific prosocial spending behavior: Does prosocial spending increase when people have been induced to make donations on previous occasions? Does prosocial spending increase when people have felt free or autonomous to donate on previous occasions and to decide on the amount of money to give? Is there an anchoring bias from an initial prosocial donation to subsequent prosocial spending? Does prosocial spending increase when people believe that helping others and being a better person is good for their own happiness?

With these questions in mind, the current research study has five main objectives that can contribute to the previous literature on prosocial spending. First, we propose that inducing prosocial spending affects subsequent behaviors. According to mere exposure research [23–25] exposure to prosocial donation facilitates subsequent prosocial spending. By contrast, dedicating the money for personal use and the absence of previous exposure to prosocial donation would both inhibit prosocial spending. Second, it is reasonable to expect that the effect of inducing prosocial spending is especially relevant when the individual has participated freely and decided on the amount. Based on self-determination theory [26], freedom in prosocial spending would satisfy psychological needs (e.g., autonomy), thus facilitating helping others because this behavior is volitional. However, compulsory donations would inhibit subsequent prosocial spending [27] Third, the anchoring bias could play a role [28]. It is common for charitable organizations and NGOs to establish a fixed amount for people’s donations. This could lead to subsequent prosocial spending that is biased toward the initial donation. Fourth, individual differences might also make a difference [29]. More specifically, we expect individuals who are high in eudaimonic happiness beliefs to spend more money in a prosocial way. Eudaimonia is an Aristotelian concept that has become a central topic in research related to well-being and happiness [30]. Eudaimonic well-being beliefs are understood here as lay people’s conceptions of what their own well-being means (contribution-to-others and self-development) [31]. It is reasonable to expect that when people base their own happiness on helping others (contribution-to-others beliefs) and being a better person (self-development), they are more predisposed to spending money to help others. By contrast, hedonic beliefs, which define personal well-being as the experience of positive emotions and the avoidance of negative experience [31] (prosocial spending may produce negative experiences associated with deception or naivety, thus inhibiting donation [32], will inhibit prosocial spending directed to helping others. Finally, we also expect autonomy in the donation decision to moderate the relationship between eudaimonic well-being beliefs and prosocial spending. The disposition towards prosocial spending in people with high eudaimonic well-being beliefs is especially reinforced when they have autonomy in the money to be given. Autonomy in decision-making describes an environment with a sense of choice and initiative [33], which seems to be the adequate context in which to translate eudaimonic beliefs into specific spending behaviors.

### Mere exposure, autonomy, and anchoring bias

The effects of prosocial behavior in general have been extensively researched [19]. In addition to the positive effects for receivers and for society as a whole, these behaviors are linked to different individual emotional and health benefits for givers, such as momentary and long-lasting well-being [34,35] and fewer depressive symptoms [36]. More specifically, prosocial spending promotes positive emotions and greater happiness [14,37]. In addition, results show that people experience emotional benefits from prosocial spending in both highly developed countries (North America and Europe) and developing countries, where the possibility of prosocial spending is reduced (e.g., some regions in Africa and Asia) [19]. Other benefits of prosocial spending are related to health and reducing cortisol levels [37].

All these positive effects of prosocial spending are incongruent with another strong human motivation: self-interest. Paradoxically, maximizing their own interests inhibits the benefits people can achieve through helping others selflessly. Therefore, it is relevant to investigate the factors that stimulate and inhibit prosocial spending. One mechanism that could be relevant is previous exposure to situations where the person displays this type of behavior. According to the mere exposure research, people are more likely to react positively to known stimuli than to novel ones [23–25] It has been proposed that previous exposure to a stimulus reduces the threat [38] and arousal associated with novel stimuli and could facilitate fluent processing [38]. In the specific case of prosocial spending, it could be proposed that the person who has been exposed to displaying this type of helping behavior will be more willing to spend money in a prosocial way on future occasions. Therefore, we propose the following hypothesis:

*Hypothesis 1: People spend more money on helping vulnerable people if they have been exposed to a prosocial donation situation than if they have not had this exposure*.

Autonomy can also play a role in the degree to which people are inclined to spend money on others in the future. Recently, prosocial spending has been related to control over the decision to help others. For instance, people experience greater well-being when the act of spending money on others is autonomous [27]. Using an experimental design, studies have shown that prosocial behavior that is autonomously motivated leads to relatively greater well-being for the helper, as well as for the recipient [27,33]. From the Self-Determination Theory [26] perspective, autonomy is a basic psychological need of humans. Autonomy (having decision-making power when performing an activity) is especially related to the positive effects of prosocial spending because individuals feel that this behavior is a consequence of their own choices [20,27,33,39].

The role of autonomy can also be critical to the prediction of prosocial behavior in general [39]. In the specific case of prosocial spending, the amount to be given can be compulsory (rules or other external factors force individuals to give a certain amount) or voluntary (the individual can decide whether to donate or not, and the amount of the donation). Autonomy describes an environment where individuals’ decisions are based on their own initiative. As Nelson and colleagues [33] pointed out, according to self-determination theory [26], autonomy in prosocial behavior satisfies three psychological needs. First, it is self-evident that *autonomy* needs are satisfied because an environment is created that stimulates a sense of choice and volition. Second, the autonomy to select the behavior satisfies the need for *competence* because the individual is capable of managing his/her own prosocial behavior. Finally, *connectedness* is also satisfied because the individual establishes a positive bond with the recipients of the aid. A situation where individuals feel free to participate in prosocial spending and decide what amount to give to help others describes a favorable environment for involvement in prosocial spending. Autonomy means that individuals’ decisions correspond to their preferences, and it facilitates positive bonds with the recipients and the prosocial spending behavior. Because autonomy is inextricably connected to intrinsic motivation [39,40], the prosocial spending is likely to continue after an initial exposure to a donation decision where there is autonomy in helping others. By contrast, forcing individuals to participate in prosocial activities could be counterproductive because they do not enjoy an activity chosen by others, which reduces their intrinsic motivation [41]. For example, compulsory programs for adolescents to stimulate their altruistic inclinations have effects that are contrary to expectations because participants are not intrinsically motivated [42,43]. Similarly, forcing individuals to spend an amount describes a less favorable environment for prosocial spending. In a compulsory donation, a positive and intrinsic bond is not stimulated in the initial exposure a donating decision, thus affecting subsequent prosocial spending. Based on these arguments, we propose the following hypothesis:

*Hypothesis 2: People spend more money on helping vulnerable people after being exposed to an autonomous donation situation than after being forced to donate a certain amount to others*.

Sometimes charitable organizations and NGOs establish fixed amounts (e.g., monthly fees) to be given by members and citizens. Fixed donations can be helpful in achieving resources to help vulnerable people, but this policy could create an anchoring bias. The anchoring bias refers to estimations that individuals make based on an initial value or information that is adjusted to yield a final decision [28]. The anchoring bias has been confirmed in different contexts, such as negotiation [44], self-efficacy [45] performance [46], and service quality evaluations [47] This bias demonstrates that individuals usually make decisions that are in consonance with previous information existing in the context. They consider the initial option or adjust their decision based on that anchor and may choose a more similar option than they would have otherwise [48]. Using visual analytics to study the anchoring effect on the decision-making process, the numerical anchor has been shown to have a significant effect on decision-making [49]. Regarding prosocial spending, the existence of fixed amounts or fees can provoke an anchoring bias that affects subsequent prosocial decisions. Accordingly, we propose the following hypothesis:

*Hypothesis 3: Initial prosocial spending characterized by a compulsory fixed amount biases subsequent voluntary prosocial donations*.

### Well-being beliefs and prosocial spending

Prosocial spending also depends on people’s personal beliefs about what well-being means to them. More specifically, the duality between hedonic vs. eudaimonic well-being beliefs can help to understand prosocial spending [31]. Hedonic beliefs, including the dimensions of “experience of pleasure” and “avoidance of negative experiences”, equate well-being with personal pleasure. When people base their own well-being mainly on pleasure, it is unlikely that they will spend money on others because they think this behavior will reduce their own positive emotions (e.g., the benefit is for others) and increase negative ones (e.g., naivety feelings). By contrast, the eudaimonic perspective defines well-being in terms of living virtuously and contributing to a greater good [50]. People with a high eudaimonic conception of their well-being are more willing to spend money on others. Although the role of hedonism is pervasive, eudaimonic beliefs are increasingly important in today’s societies. In fact, “modernization is evolving into a process of human development” [51], which emphasizes human autonomy, creativity, and self-expression. Compared to other periods in our history, we now live in a highly civilized society [52] where an increasing number of people are economically secure, characterized by a shift from giving priority to economic and physical security to emphasizing self-expression and quality of life. The change in values and beliefs highlights personal self-development and empathy toward others, making people more aware of others and more willing to help those in need [53]. In this context where life has reached a higher meaning than merely acquiring material possessions, eudaimonic well-being beliefs have become very relevant.

Eudaimonia is a philosophical concept introduced by Aristotle [54]that refers to a life lived to its fullest potential [55]. According to McMahan and Estes [31], eudaimonia helps to understand the beliefs that lay people have about the definition of their own well-being. These authors differentiate between two types of eudaimonic well-being beliefs: self-development beliefs (degree to which individuals believe that their own well-being is based on personal growth and being a better person) and “contribution-to-others” beliefs (degree to which individuals believe that their own well-being is based on helping others).

The well-being beliefs perspective [31] is especially relevant for the current research study because it refers to individual differences that can guide prosocial spending behaviors. Personal beliefs, in general, reflect stable views about the reality that activate motivational goals [56] and act as a cognition that filters information-processing and has an impact on subsequent behaviors [57]. Individuals orient their behaviors and the search for aspects in their environments based on their beliefs. Transferring this argument to the *eudaimonic way of life* [30], individuals who are high in eudaimonic well-being beliefs are likely to display behaviors that allow *doing* good [58]. When individuals believe that their happiness is based on helping others (contribution-to-others beliefs), personal growth, and being a better person (self-development beliefs), they are willing to display behaviors that help others, including prosocial spending directed to helping vulnerable people. The connection between contribution-to-others beliefs and prosocial spending is evident. When individuals interpret that living a purposeful life involves helping others, greater prosocial spending is likely. Similar endeavors have shown that benevolence, as an individual factor, is causally related to cooperation with others [59], or that empathy induction increases helping behavior [10]. Regarding self-development beliefs, it is difficult to imagine that a person can fulfill his/her potential and achieve a virtuous life without showing concern for the problems of other people, especially those who are vulnerable. Based on these arguments, we propose the following hypothesis:

*Hypothesis 4: Eudaimonic well-being beliefs (contribution-to-others and self-development) are positively related to prosocial spending*.

### The moderation role of autonomy

According to person x situation interactionism [60], the interaction between stable individual differences and situational factors allows a better understanding of human behavior. Some situations are favorable to certain personal characteristics, stimulating behaviors that are congruent with these relatively stable traits (e.g., people high in extraversion enjoying social activities). This rationale can also be useful in predicting prosocial spending. As described above, individuals with high eudaimonic well-being beliefs are likely to spend more money on vulnerable people than individuals with low beliefs. In addition, the existence of an autonomy-supportive context seems to be the optimal *breeding ground* for prosocial spending behavior in people with high eudaimonic well-being beliefs. Autonomy matches the predisposition of these individuals to help others. Taking into account the postulates of self-determination theory [26,33], it is reasonable to expect that individuals high in eudaimonic beliefs would be especially sensitive to the satisfaction of needs provided by autonomy: they would take the initiative to help others in a way that is congruent with their eudaimonic beliefs (autonomy); they would manage the desired prosocial behavior themselves (competence); and they would achieve a positive bond with the recipients of the help, facilitating the expected virtuous life (connectedness). After a first exposure to a donation decision involving autonomy, individuals high in eudaimonic beliefs will be especially willing to spend money on others on future occasions. Therefore, we propose the following hypothesis:

*Hypothesis 5: Autonomy moderates the relationship between eudaimonic well-being beliefs and prosocial spending, such that this relationship is stronger after an autonomous donation situation than after individuals are forced to donate a fixed amount to others*.

## Methodology

### Ethics statement

The study was conducted in accordance with the Declaration of Helsinki and it was evaluated and approved by the Ethical Committee of the University of Valencia. All participants were briefed about the objectives of the study and gave their written and informed consent on the experimental procedure where anonymity and confidentiality were guaranteed by the researchers. In addition, participants were informed that they were free to leave the study at any time or prevent the use of the data they provided.

### Participants and procedure

To determine the number of participants, we followed the rule suggested by Sekaran and Bougie [61]. Accordingly, we considered more than 10 individuals for each variable (see Table 1) included in the research study. We tested the statistical power of the sample with G*Power software. Based on the R^2^, a sample size of 200 participants, an alpha error probability of .05, and 7 predictors (control variables, predictors, and interaction), the results showed a power greater than .95, indicating excellent predictive power.

**Table 1:** Control/Experimental groups, steps in the procedure, and instructions.

	Control 1 (C1) “Totally Self-focused”	Control 2 (C2) “No exposure”	Experimental 1 (E1) “Compulsory fixed donation”	Experimental 2 (E2) “Compulsory donation”	Experimental 3 (E3) “Autonomous donation”
Step 1	Welcome and signing of the informed consent document				
Step 2	Completing the questionnaire				
Step 3	Voucher Self-focused. Information about NGO was omitted	No voucher This step was omitted (no exposure)	Compulsory donation to the NGO (two euros)	Compulsory donation to the NGO, but no amount specified	Freedom to donate to the NGO
Step 4	10’-break				
Step 5	Final decision. Independently of the previous option, all participants were asked to distribute the 10 euros (voucher value) among the three options: Personal use at the university store Personal use at the cafeteria Donation to an NGO				

A total of 200 undergraduate university students were randomly distributed into five groups. 74.1% of the participants were male and 24% female, and the mean age was 22.07 years (SD = 5.45). There were three experimental groups where the focus was on a first exposure to prosocial spending directed to helping vulnerable persons through an NGO. For the first experimental group (“compulsory fixed donation” N = 40), the donation was compulsory, and the amount of money was fixed. For the second experimental group, the donation was compulsory, but there was no fixed amount (“compulsory donation” N = 40). Finally, participants in the third experimental group (“autonomous donation”, N = 40), were free to donate or not, and they chose the amount to be given. After participants had been welcomed, they signed an informed consent document to participate in the study. In the second step, participants were asked to complete a questionnaire about their well-being beliefs and provide information about age and sex. In the third step, and after answering the questionnaire, participants received a voucher with a value equivalent to 10 euros as compensation for their participation in the study. Participants never received actual physical money, but instead they were given a voucher for personal use. Participants in the first experimental condition (“compulsory fixed donation”) were informed that, compulsorily, two of the 10 euros (20%) would go to an NGO to help vulnerable people. This amount (two euros) was determined because it is a significant but not extremely large amount, and it simulated NGOs’ usual fee policies to some extent. The rest of the money (8 euros, 80%) would be for personal use, and each participant could spend it on any product from the university store (pencils, notebooks, etc.). Participants in the second experimental condition (“compulsory donation”) were informed that part of the 10 euros *must* go to an NGO to help vulnerable people, but the amount was chosen by the participants. The rest of the money would be for personal use, and each participant could spend it on any product from the university store (pencils, notebooks, etc.). Finally, participants in the third experimental group (“autonomous donation”) were informed that they could freely decide whether to donate or not, and they could choose what part of the 10 euros, if any, they wanted to give to the NGO. The donation could vary from 0 to 10 euros. They also decided on the amount for personal use. The fourth step consisted of a 10-minute break. After this break, the fifth and final step involved the participants’ final decision about the 10 euros they received for participating in the study. They were informed that “participants have received different instructions during the process, and the research team’s plan is to offer the same final options to all of them for the definitive distribution of the 10 euros”. Therefore, all participants received a total of 10 euros (voucher). All of them were free to redistribute the 10 euros in another way. To do so, they had three alternatives: a) personal use at the university store; b) personal use at the cafeteria; and c) a donation to the NGO. Each of the three amounts could vary from 0 to 10 euros, but in all, it had to add up to 10 euros. Participants were informed that their decision would be respected, and that the money they decided on would be sent to the NGO that helps vulnerable persons.

There were two control groups. Participants in the first control group (“totally self-focused”, N = 40) followed the same scheme designed for the experimental groups. The only difference was in the third step (first exposure). In this step, participants received a 10-euro voucher as compensation for their participation in the study. They were informed that this amount was for personal use, and each participant could spend it on any product from the university store (pencils, notebooks, etc.). Therefore, participants in the first control group could not donate any amount to the NGO in the third step. In fact, they did not receive any information about NGOs. The second control group (“no first-exposure”, N = 40) followed the same scheme as the experimental groups, but the third step (first exposure) was omitted, and they went directly to the break (information about the voucher was omitted and it only appeared at the final decision). In the last step (final decision), participants in the second control group also received a 10-euro voucher as compensation for their participation in the study, and they decided on the distribution of the amount using the same instructions as the other participants in the study: a) personal use at the university store; b) personal use at the cafeteria; and c) a donation to the NGO. We used this format with these three alternatives in the last step for all the participants because it was different from any of the previous formats (new to everyone), but it still contained the option of donating to an NGO. See Table 1 for an overview of the control and experimental groups and their first exposure situation and subsequent donation behaviors.

### Variables

Eudaimonic well-being beliefs were measured by using the “Beliefs about Well-being Scale” by McMahan and Estes [31]. To focus the attention on well-being beliefs, respondents were asked to indicate their level of agreement about the contribution of each facet (item) to well-being in their own lives: “The experience of well-being and the good life necessarily involves…”. *Contribution-to-others* beliefs were assessed using 4 items, with responses scored on a 7-point scale ranging from 1 (strongly disagree) to 7 (strongly agree). A sample item includes: “Living in a way that benefits others”. Cronbach’s alpha for the scale was α = .85. *Self-development* well-being beliefs were measured using 4 items, with responses ranging from 1 (strongly disagree) to 7 (strongly agree). A sample item was: “Working to achieve one’s true potential”. Cronbach’s alpha for the scale was α = .75.

We also measured hedonic well-being beliefs. McMahan and Estes [31] proposed these beliefs to capture the other way individuals interpret their own well-being. According to hedonic beliefs, well-being is equivalent to personal pleasure. We controlled for hedonic beliefs in order to have a more solid test of the specific or differential role of eudaimonic beliefs in predicting prosocial spending. Hedonic beliefs were measured using the “Beliefs about Well-being Scale” by McMahan and Estes [31]. Again, respondents were asked to indicate their opinion about the contribution of each facet (item) to well-being in their own lives. *Experience of pleasure* was measured using 4 items, with responses ranging from 1 (*strongly disagree*) to 7 (*strongly agree*). A sample item was: “Experience euphoria and pleasure”. Cronbach’s alpha for the scale was α = .81. The second hedonic dimension, *avoidance of negative experience*, was assessed using 4 items, with responses ranging from 1 (*strongly disagree*) to 7 (*strongly agree*). A sample item was: “Not experience hassles”. Cronbach’s alpha for the scale was α = .85.

*Prosocial spending* was assessed considering the participants’ final decision (fifth step). Therefore, prosocial spending was operationalized as the number of euros given to the NGO to help vulnerable persons As mentioned above, in this final decision all participants should distribute the 10 euros they received for participating in the experiment among three purposes: a) personal use at the university store; b) personal use at the cafeteria; and c) donation to the NGO. The total amount had to correspond to 10 euros. Our measure reflects the real money given to the NGO.

Because both gender [11] and age [62] have been shown to have a possible effect on the degree to which people behave in a prosocial manner, we have included them as control variables.

## Results

### Preliminary results

Means, standard deviations, Cronbach’s alpha coefficients, and the correlations among the study variables are displayed in Table 2.

**Table 2:** Means, standard deviations, inter-correlations, and Cronbach’s alpha of the scale (between parentheses).

	Construct	M	SD	1	2	3	4	5	6	7
1.	Sex			—						
2.	Age	22.07	5.45	.09	—					
3.	Experience of pleasure	5.38	.90	-.27	-.20**	—	*(*.*83)*			
4.	Avoidance of negative experience	3.83	1.42	-.05	.03	.11	—	*(*.*85)*		
5.	Contribution-to-others	5.33	.95	.04	.08	.17*	.00	—	*(*.*85)*	
6.	Self-development	5.90	.83	-.01	.07	.37**	-.07	.38**	—	*(*.*75)*
7.	Prosocial spending	3.90	3.88	.00	-.01	-.10	-.29**	.17**	.20**	—

Note: N Listwise = 192,

* *p* < .05,

** *p* < .01

Considering scores of standard deviations, there was low dispersion in some variables: experience of pleasure, contribution-to-others, and self-development. By contrast, dispersion was higher in avoidance of negative experience. Values corresponding to Cronbach’s alphas were above .75 for all the study measures. As expected, there was a significant positive relationship between contribution-to-others and self-development well-being beliefs. Moreover, these eudaimonic well-being beliefs, measured before manipulation, were positively related to the final prosocial spending (number of euros given to the NGO in the last step). By contrast, the significant link from avoidance of negative experiences (hedonic beliefs) to prosocial spending was negative, whereas the relationship between experience of pleasure (hedonic belief) and prosocial spending was not significant. Finally, there were significant positive links from experience of pleasure to both dimensions of eudaimonic well-being beliefs (“contribution-to-others” and self-development).

### Hypothesis testing

To determine whether baseline condition differences existed prior to the manipulation, a multivariate analysis of variance was performed. As expected, the five groups did not differ on their contribution-to-others well-being belief levels (*F*_(4, 195)_ = 1.48, *p* > .05), or on their self-development beliefs (*F*_(4, 195)_ = 1.17, *p* > .05).

**Fig 1:**
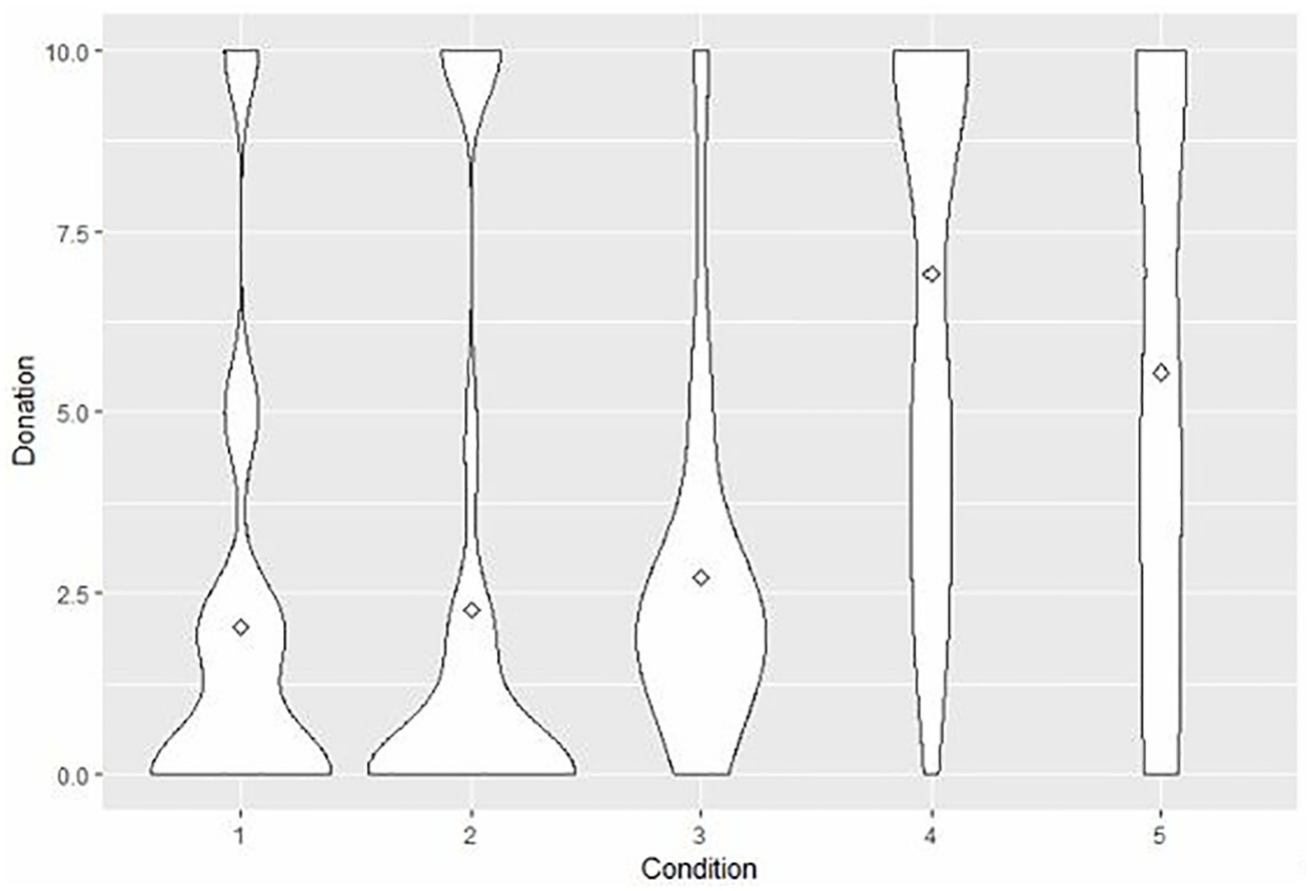
Distribution differences in prosocial spending. Note: N group = 40; 1 = “totally self-focused” (*M* = 2.03, *s*.*e*. = .49), 2 = “no first-exposure” (*M* = 2.28, *s*.*e*. = .59), 3 = “compulsory fixed donation” (*M* = 2.73, *s*.*e*. = .40), 4 = “compulsory donation” (*M* = 6.90, *s*.*e*. = .54), 5 = “autonomous donation” (*M* = 5.55, *s*.*e*. = .60).

Before testing the differences between the groups’ means on donation, we tested the three assumptions of one-way analysis of variance. In our case, both the assumption of normal distribution and heterogeneity of variance were violated. Therefore, we used the non-parametric test for independent groups: the Kruskal-Wallis test. The results revealed significant differences in the amount of prosocial spending across the five groups *χ*^2^ (4) = 56.87, *s*.*e* = 12.58 *p* < .001. Furthermore, post-hoc analyses were performed, and Bonferroni correction for multiple tests was applied, adjusting significance. The results showed that the amounts of money corresponding to both the “compulsory donation” (E2) and “autonomous decision” (E3) conditions were significantly higher than those corresponding to the other three conditions: the two control groups (“self-focused”-C1 and “no exposure”-C2) and the “compulsory fixed donation” group (E1) (see Fig 1). For conditions C1, C2, and E1, donations tended to concentrate on 2 and 2,5 euros. By contrast, donations were distributed in a more balanced manner for conditions E2 and E3.

To test H1 (the effect of previous exposure), we examined the differences between the two control groups (“self-focused”-C1 and “no exposure”-C2), on the one hand, and E2 (“compulsory donation”) and E3 (“autonomous donation”), on the other. The amounts of money donated by participants who were not exposed to a previous donation situation (C1 and C2) were significantly lower than for those who were previously exposed to both E2 (*χ*^2^ = -55.28, *p* < .05; *χ*^2^ = -54.17, *p* < .05, respectively) and E3 (*χ*^2^ = -75.80, *p* < .05; *χ*^2^ = -74.58, *p* < .05, respectively). Therefore, H1 was supported.

Regarding H2 (effect of autonomy), we paid attention to the comparison of E2 (“compulsory donation”) and E3 (“autonomous donation”), which did not reveal significant differences (*χ*^2^ = -20.41, *p* > .05). Accordingly, the effect of autonomy was not significant, and H2 was not supported.

Our H3 tests the anchoring bias. To do so, we focused on the comparison of the two experimental groups where compulsory donation existed: E1 (where an anchor of 2 euros was used) and E2 (donation was compulsory, but without a fixed amount or anchor). The donated amount was higher for E2 than for E1 (*χ*^2^ = -52.14, *p* < .05). The anchor limited the amount of money donated in the final decision, confirming H3 (see Fig 1).

To test H4 and H5, we computed a hierarchical regression analysis (Table 3). The variables were mean centered before introduced in the regression. In the first step, the control variables were introduced and they explained 9% (*ΔR*^*2*^ = .09, *p* < .01) of the total variance of prosocial spending. In the second step, the two eudaimonic wellbeing beliefs and the experimental condition were added as predictors, and they explained a significant increase of 9.4% (*R*^*2*^ = .18, *ΔR*^*2*^ = .09, *p* < .01) of the total variance of prosocial spending. In the third step, we introduced the two interaction terms, between contribution-to-others and “autonomous donation”, and self-development and “autonomous donation”. Both interactions accounted for a significant proportion of the variance in prosocial spending (*R*^*2*^ = .22, *ΔR*^*2*^ = .03, *p* < .05).

**Table 3:** Summary of regression analysis.

*Variable*	*β*	*s*.*e*.
Constant	4.79**	3.83
Age	-.04	-1.02
Sex	-.12	-.20
Experience of pleasure	-.59*	-2.09
Avoidance negative experience	-.98**	-3.86
Contribution-to-others	.48*	1.66
Self-development	.42	1.34
Autonomy	1.34*	2.07
Contribution-to-others x Autonomous decision	-.53	-.67
Self-development x Autonomous decision	2.14**	2.61
*RMSE*	3.51	

*p < .05.

**p < .01 (one tailed)

Therefore, H4 was confirmed, but only for contribution-to-others beliefs. Contribution-to-others beliefs had a positive and significant direct link to prosocial spending (*β* = .48, *s*.*e*. = .29, *p <* .05). However, this link was not significant for self-development (*β* = .42, *s*.*e*. = .31, *p >* .05). Interestingly, the results also showed that both experience of pleasure (*β* = -.59, *s*.*e*. = .28, *p* < .05) and avoidance of negative experiences (*β* = -.98, *s*.*e*. = .25, *p* < .05) (hedonic well-being beliefs) had a significant negative relationship with prosocial spending. The strong association between avoidance of negative experiences and prosocial spending is especially noteworthy (see Table 3).

The moderation role of autonomy in the relationship between eudaimonic beliefs and prosocial spending (H5) was confirmed, but only for self-development beliefs (see Table 3). Because the moderator variable we propose in this model (autonomy) is dichotomous, we created a dummy variable where 1 was assigned to participants who had autonomy in the first exposure (E5) and 0 was assigned to the rest of the participants.

**Fig 2:**
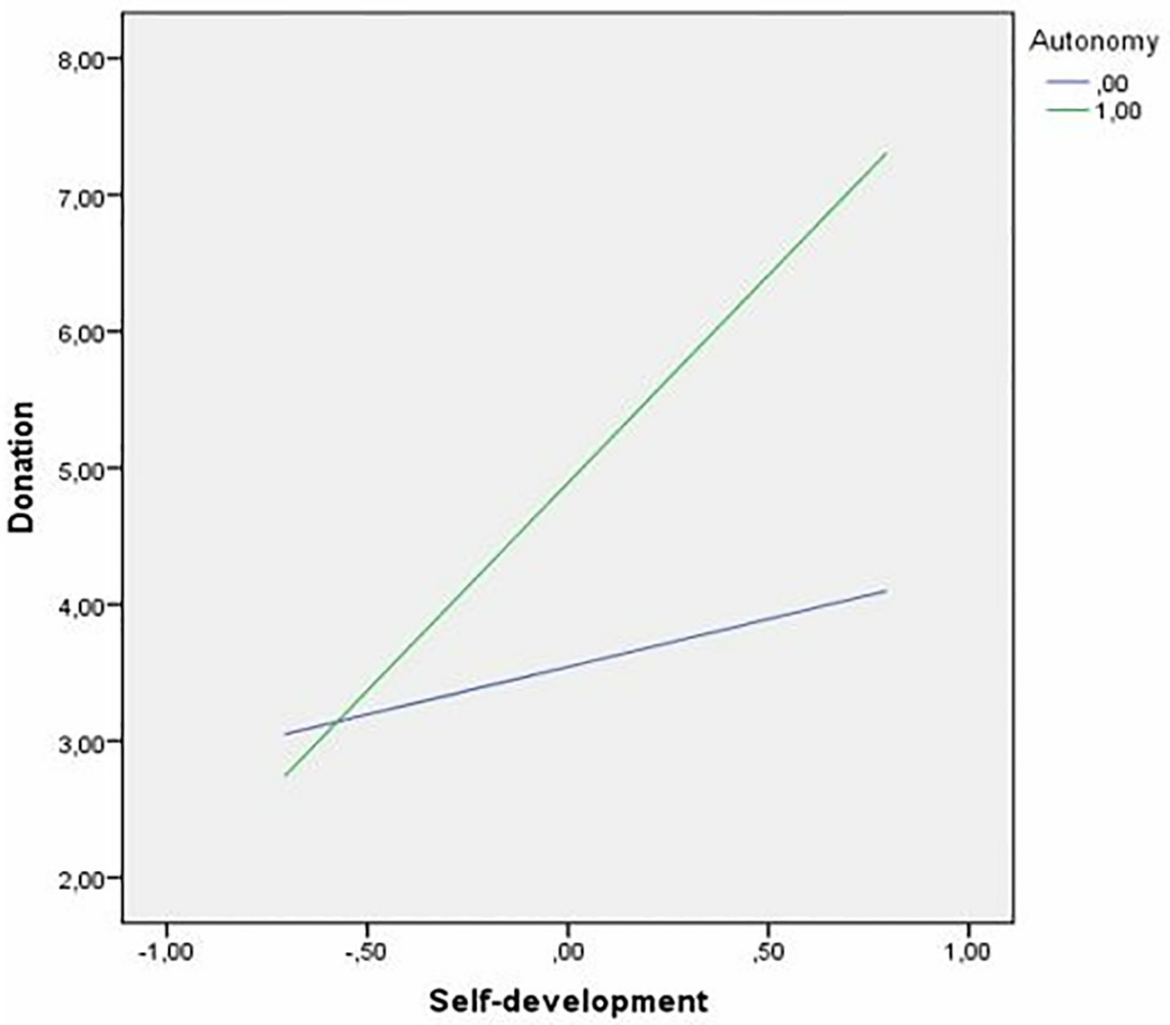
Moderation of autonomy in the relationship between self-development beliefs and prosocial spending.

The graphical representation of the interaction confirmed that autonomy strengthened the positive relationship between self-development and prosocial spending (see Fig 2).

## Discussion

The current experimental study had five main goals. First, we examined the prosocial spending decision after participants had been exposed to a donation to an NGO. In this regard, we compared the amount of money donated by participants in two experimental conditions (“compulsory donation” vs. “autonomous donation”) vs. two control conditions (“no first-exposure” and “totally self-focused”). Our results confirmed the hypothesis that exposing individuals to prosocial spending affects subsequent behaviors: individuals who participated in “compulsory donation” and “autonomous donation” gave more money to the NGO than participants in the two control groups. Second, we examined whether autonomy in decision making stimulates prosocial spending. Our results showed that autonomy in decision-making does not influence prosocial spending. In fact, money given by participants in the “autonomous donation” group was not statistically different from the money given by individuals in the “compulsory donation” group. Third, we explored the existence of an anchoring bias. According to our findings, the anchoring bias exists in prosocial spending. Participants in the “compulsory fixed donation” group made a final decision that is biased toward an initial compulsory and fixed amount, limiting their prosocial spending. Fourth, we investigated the role of eudaimonia well-being beliefs, observing the existence of a significant positive direct link from contribution-to-others beliefs (measured before manipulation) to the final NGO donation decision. Finally, we investigated how autonomy in prosocial spending decisions moderates the relationship between eudaimonic well-being beliefs and money donated to the NGO. Our findings indicate that autonomy strengthens the link from self-development well-being beliefs to the donation decision.

These findings make a series of contributions that expand the knowledge about prosocial spending behavior. The positive outcomes of prosocial spending have been widely investigated [14,18–20,27]. However, little is known about the factors explaining this behavior. According to our results, there is a strong mere exposure effect. It is well-known that mere exposure to a stimulus (i.e., prosocial spending) tends to create positive reactions to this familiar stimulus on subsequent occasions [23–25]. The current research study confirmed that this effect also appears in prosocial spending. In addition, this mere exposure effect was quite consistent because it was observed not only in autonomous decisions (“autonomous donation”), but also when participants were forced to donate to an NGO in the first exposure (“compulsory donation”). Therefore, autonomy was not a direct significant precursor of prosocial spending. The possible supportive context of autonomy associated with prosocial spending was not confirmed. In other words, mere exposure to prosocial spending facilitates subsequent donations to an NGO, even if individuals are forced to donate an amount in the initial decision. By contrast, the anchoring bias [28]was able to change the amount donated to an NGO in those who were exposed to a fixed amount of prosocial spending (“compulsory fixed donation”). We established an initial small compulsory amount (2 euros) that biased the subsequent free decision and significantly reduced the amount given to the NGO (see Fig 1). In fact, in their final decision, these people gave less than half the money donated by other participants who were also initially exposed to prosocial spending but without a fixed amount (“autonomous donation” and “compulsory donation”).

Eudaimonic well-being beliefs have an additional role in predicting prosocial spending. Individuals differ in the degree to which they interpret that their own well-being is based on helping others (“contribution-to-others”), personal growth, and being a better person (self-development) [31]. Although hedonism (maximization of pleasure and avoidance of negative experiences) is a strong motivator of behavior, humans have been able to create complex bonds and interactions in our societies, where solidarity and helping others are present [5]. Individuals are also capable of defining their well-being as a virtuous way of life where efforts to improve self-development and be a good person are necessary. Our findings revealed that eudaimonic contribution-to-others beliefs are positively related to prosocial spending directed to an NGO to help vulnerable persons. These beliefs reflect a cognition where one’s own happiness is associated with helping others, facilitating behaviors of spending money on others. Interestingly, one of the hedonic beliefs (avoidance of negative experiences) had a strong significant relationship with prosocial spending, but in the opposite direction. That is, individuals who defined their own well-being as the avoidance of negative emotions tended to reduce prosocial spending. A tentative explanation for this result would be the association between avoidance of negative emotions and conservative behavior that avoids possible deceptions, overconfidence, or naivety related to spending on others. This issue should be investigated in future studies. In any case, although it was not our main focus, this negative relationship between avoidance of negative experiences and prosocial spending is quite relevant due to the negative bias in human behavior. In their reviews, Baumeister, Bratslavski, Finkenauer, and Vohs [63], Rozin and Royzman [64] clearly confirmed that bad events have greater and more lasting effects on human behaviors than good ones in different contexts. Baumeister and colleagues [63] interpreted this negative bias as an adaptive mechanism because it allows humans to avoid terrible events that could threaten survival. Transferring this argument to the aforementioned result, defining one’s own well-being as avoidance of negative experiences could inhibit prosocial spending because the focus could be on possible negative experiences of this behavior (e.g., feeling naïve).

According to person x situation interactionism [60], we observed a significant interaction between self-development well-being beliefs and autonomy of decision-making in predicting prosocial spending. Individuals who define their own well-being based on personal growth and being a better person are willing to spend money on the ONG, but this is especially evident when they are in a context that supports autonomy. Consistent with Self-Determination Theory [26,33], this autonomy allows these individuals to take the initiative to help others, reflecting a sense of choice and volition that is congruent with their eudaimonic beliefs, which facilitates well-being as personal growth and a virtuous life. Unexpectedly, the interaction was not significant for the other dimension of eudaimonic beliefs: contribution-to-others. It is possible that this difference between the two dimensions of eudaimonic well-being beliefs (one of them moderates and the other does not) is based on their somewhat distinct nature. As Pătraş, Martínez-Tur, Estreder, Gracia, and Moliner [65] argued, contribution-to-others beliefs are cognitions oriented towards other people. In the current study, this orientation towards others is achieved through prosocial spending. The main focus is on helping others, regardless of the existing level of autonomy. By contrast, self-development beliefs orient cognition toward internal personal growth [65]. This personal self-development cannot be achieved if the individual is forced to act in a pre-established direction and does not have the feeling that his/her decision is based on personal initiative and choice. Of course, this is a tentative hypothesis that could be confirmed in future research studies. We also proposed that gender and age would affect prosocial behavior, coinciding with previous studies [10]. However, the results showed that their effects are not significant. A tentative explanation for this finding could be related to the fact that both males and females behave equally when the context is experimentally neutral, and there is no “social frame” in the experimental setting [66]. The experimental neutral induction toward prosocial spending might also explain that the age of the participants had no effect on the extent of helping behavior, again highlighting the importance of the social context [67].

Our findings also have practical implications in at least three ways. First, exposure to prosocial spending seems to be a very good strategy, even if giving economic aid is not compulsory. There are different contexts where citizens could be exposed to opportunities for prosocial spending (e.g., schools, workplaces, associations). Second, the practice of establishing fixed donations (e.g., monthly fees) to NGOs can ensure incomes for vulnerable people and other initiatives, but the anchoring bias should also be considered. Fixed amounts could create a reference that remains stable, in terms of the money the person will donate over time. Third, if we assume that prosocial spending is positive for individuals (for both givers and receivers) and for society in general, our results advise against social communication that could facilitate a hedonic interpretation of happiness. The messages that are sent to citizens, for example through the media (television, internet, etc.), usually equate well-being and happiness exclusively with hedonism. However, other messages are possible where happiness is based on eudaimonic beliefs.

Although this study makes a number of theoretical and practical contributions to the research in the area, it also has limitations that can be reduced in future investigations. First, participants in the current study were students in controlled settings. In order to achieve a richer picture, we suggest that further research also consider real settings, for example, using members of NGOs to answer the research questions. Second, people playing different roles in society (e.g., offering human services vs. commercial activities) can also have distinct beliefs and respond to autonomy contexts in a very different way. Therefore, these differences should also be considered in further research. Third, based on the research study by Vohs and colleagues [68] it is reasonable to expect that priming people with the idea of money will make them less likely to help others in need or donate to charity. Although our participants received a voucher rather than physical money, future studies could examine the possible differential effects of using physical money vs other types of compensation. Fourth, although the way we provided autonomy and forced the donations was objective and clear, the measurement of perceptions could be considered in future studies. Assessing perceptions of autonomy and donation obligation can have at least two important functions: a) to test how participants interpret the manipulation and whether it is transferred to the decision-making; and b) to check whether the manipulation works in terms of participant perceptions. Fifth, it is possible that participants have shown behaviors to help an NGO in the past, which could influence their decisions during the experiment. Future studies could clarify and control the role of this previous experience. Sixth, the social distance between the giver and the receiver might influence the quantity of the money donated [69]. Therefore, the fact that the receiver of the prosocial spending in our experiment (NGO) was an intermediary organization, and not the people in a vulnerable situation directly, might reduce the extent of the donation. Accordingly, we propose that further studies could consider both NGOs and direct recipients of the prosocial spending. Finally, another limitation of this study might be related to the limited temporal perspective. Even though we are using an experimental study design, and well-being beliefs tend to be stable over time, we suggest an experience-sampling design for further research, in order to closely study prosocial spending behavior and its variations over time.

In spite of these limitations, the current research study contributes to knowledge by exploring critical factors that explain money spent on NGOs. Both contextual and individual differences play a role. On the one hand, the exposure to prosocial spending and the anchoring bias reflect conditions that stimulate and/or inhibit donations. On the other hand, when individuals define their own well-being based on helping others, they are more willing to spend money on NGOs. These findings offer insights about how to achieve a society with greater solidarity by promoting prosocial spending in the different contexts and a change in well-being beliefs beyond hedonism.

## Supporting information

S1 Dataset (XLSX)
